# Voice hoarseness in a patient with underlying Eisenmenger’s syndrome: a case report

**DOI:** 10.1186/s40463-019-0358-3

**Published:** 2019-08-19

**Authors:** Palak Suryavanshi, Sreejit Parameswaran, Nishant Sharma, Russell A. Murphy, Anil R. Sharma

**Affiliations:** 1 College of Medicine, University of Saskatchewan, Saskatoon, SK Canada; 2 Division of Otolaryngology – Head and Neck Surgery, Department of Surgery, University of Saskatchewan, 4th floor, Suite B419, 107 Wiggins Road, Saskatoon, SK S7N 5E5 Canada

**Keywords:** Eisenmenger syndrome, Patent ductus arteriosus, Hoarseness, Recurrent laryngeal nerve, Pulmonary arteries

## Abstract

**Background:**

The natural history of patients diagnosed with Eisenmenger’s Syndrome typically revolve around the pediatric population. Medical advances have allowed these patients to live longer and present with a different subset of symptoms as a result of the progression of their disease process.

**Case presentation:**

In this case report, we discuss a 77-year-old Caucasian female with Eisenmenger’s Syndrome presenting with hoarseness. Clinical and imaging studies reveal a left vocal cord paralysis secondary to a progressively enlarging patent ductus arteriosus (PDA) and dilation of pulmonary arteries causing mass effect on the left recurrent laryngeal nerve.

**Conclusion:**

From a clinical perspective, this case highlights the need for otolaryngologists to be aware of the pathophysiology of Eisenmenger’s Syndrome as it progresses with age.

## Background

Eisenmenger Syndrome is a congenital heart disease which presents with a large cardiac defect (ventricular septal, atrial septal defect or patent ductal arteriosus [PDA]) and an overriding aorta [1]. Due to this defect patients develop pulmonary arterial hypertension (PAH) causing a central reversal right-to-left shunting of the heart. It is reported that the mean life expectancy of patients with Eisenmenger pathology is 32.5 ± 14.6 years [2]. In fact, there is a four-fold increase in mortality among patients with Eisenmenger syndrome [3], when compared to the general population. Often patients who survive the Eisenmenger syndrome with PAH into middle and old ages, have long terms effects such as cyanosis, polycythemia, renal dysfunction, and poor exercise tolerance [4].

In clinical practice, PDA is the most common type of extracardiac shunt found in patients [5]. PDA accounts for approximately 10–12% of all congenital heart defects in the adult population. Women have a higher incidence of PDA compared to men [5]. A patient with patent ductus arteriosus leading to the production of hoarseness of voice due to left recurrent laryngeal nerve paralysis was described by Fetterolf and Norris in 1911 [6] and Nakahira, Nakatani and Takeda [7]. These authors stated that PDA might result in pulmonary artery dilatation causing left recurrent laryngeal nerve paralysis. Usually hoarseness associated with PDA is historically present at birth [8]. Recurrent laryngeal nerve paralysis causing hoarseness secondary to a mass effect is an uncommon complication [9, 10]. These complications have been recorded in literature, however are rarely seen by an Otolaryngologist in the community for a patient presenting with hoarseness.

Due to this occurrence being a rare phenomenon, we present a case of left vocal cord immobility in a 77- year-old woman with Eisenmenger Syndrome secondary to a large PDA in which CT was able to depict a mass effect. Informed consent has been obtained from the patient for this case study.

## Case presentation

In this case report we present a 77-year-old Caucasian woman with a 4–5 months history of progressive hoarseness and dyspnea. The patient has a long-standing history of PDA, which was identified at birth. Patient’s past medical history is significant for a cardiac catheterization study revealing a bidirectional shunting through a PDA resulting in severe PAH diagnosed in 1970s. Other medical history includes advanced COPD with home oxygen, gastroesophageal reflux disease, essential hypertension, diabetes mellitus type II, an episode of transient cerebral ischemic attack and secondary polycythemia (between years 1986–2018, high hemoglobin levels were observed – 170.3 ± 3.3 g/L).

**Fig. 1:**
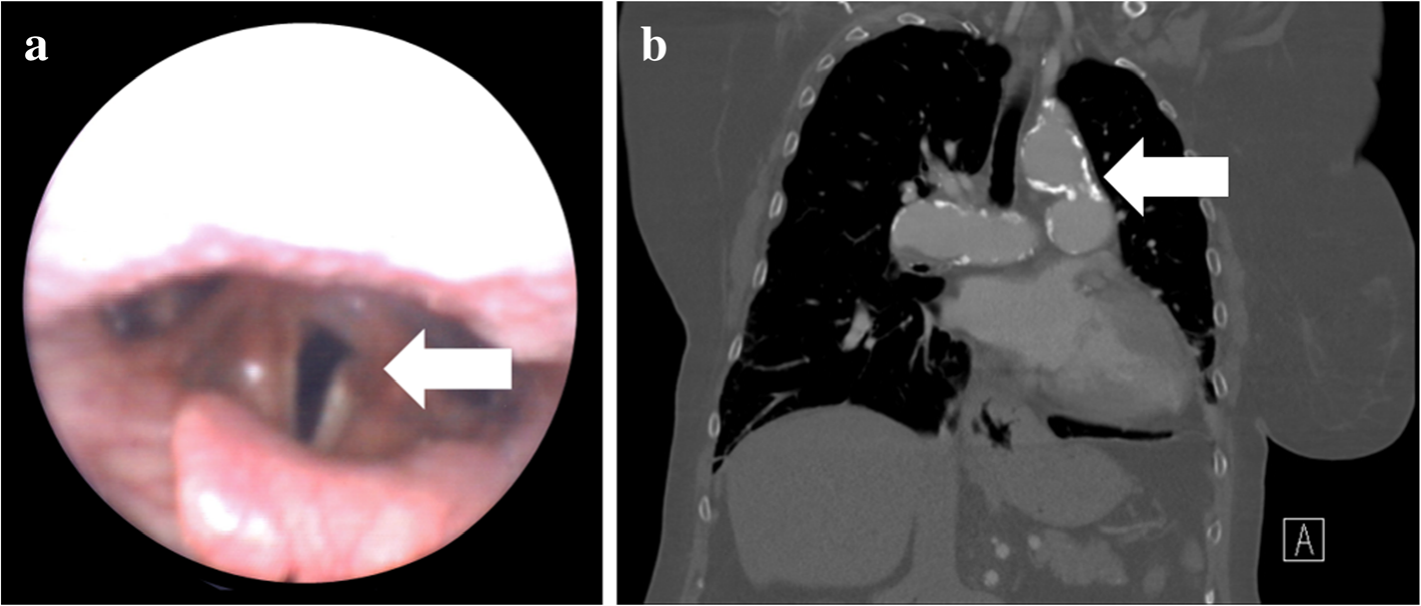
Physical examination and investigations, **a**) Visualization of the vocal cords by a flexible nasopharyngeal scope. Left immobile vocal cord seen in paramedian location; **b**) Coronal CT image showing a large partially calcified PDA with enlarged central pulmonary arteries responsible for putative mass effect and the Eisenmenger pathologies

On examination, the patient had increased breathiness to her voice quality and a left vocal cord paralysis was identified on a nasopharyngeal scope (Fig. 1 a). A CT scan was organized to follow the course of the left recurrent laryngeal nerve. The CT scan showed a large, partially calcified PDA, with associated PAH, and enlarged central pulmonary arteries (Fig. 1 b). It was concluded that the vocal cord paralysis was secondary to an enlarged PDA as a sequelae to her underlying Eisenmenger pathology resulting in a mass effect. Regarding the left vocal cord immobility, her paralyzed vocal cords were paramedian allowing for some voice and airway protection.

It is anticipated that this will be a chronic issue for the patient since her underlying syndrome has been present for decades. Due to missing patient records, information on why the patient never underwent surgery for PDA remains unknown. Given her comorbidities, the patient was determined unfit for surgery. To verify, the patient was referred to a cardiothoracic surgeon who deemed her inoperable as well. Follow up at as needed basis was arranged with the patient.

## Discussion

The recurrent laryngeal nerves are branches of the Vagus nerve which carry motor, sensory, and parasympathetic fibres and supply the muscles involved with speech articulation and vocalization. The left recurrent laryngeal nerve continues on and loops around the arch of aorta. It sits in the triangle formed by the aortic arch, ligament arteriosum and the pulmonary artery. Due to this delicate anatomical course and the triangle being 4.0 mm wide, the left nerve is vulnerable at this point [6]. It was reported that in unilateral thoracic pathologies, left-sided recurrent laryngeal nerve injuries are 1.75 times more common than right-sided injuries [8]. Numerous other diseases in the head, neck, and thoracic regions can cause recurrent laryngeal nerves injuries including inflammatory, neoplastic, cerebrovascular, heart, and degenerative processes.

There are rare cases that describe a PDA leading to hoarseness of voice [6, 7]. Fetterolf and Norris in 1911 explained the anatomical reason for a PDA to cause left recurrent laryngeal nerve paralysis [6] and Nakahira and colleagues [7] described a similar case study where PDA was causing left recurrent laryngeal nerve paralysis as well. Fetterolf and Norris concluded that in order for left recurrent laryngeal nerve paralysis to happen it is mandatory for the nerve being compressed to be located either between the left pulmonary artery and the aorta or the aortic ligament [6]. It has been reported to be an unusually rare phenomenon. Other reports have shown vocal cord paralysis secondary to PAH [11], left ventricular failure [12] and cardiovocal syndrome [8]. Nakahira and colleagues [7] refuted that if the majority of vocal cord paralysis happened due to dilated pulmonary arteries there would be an increase in the number of people with vocal cord paralysis and that is not the case. There are numerous etiologies suggested such as enlarged lymph nodes, pleural effusion and pericarditis which can also cause vocal cord paralysis [13], yet the exact mechanism is not yet determined.

For our patient, PDA was recognized earlier in the patient’s life and yet it did not produce any voice symptoms until her late 70s. The patient became gradually symptomatic over 4–5 months prior to seeing an otolaryngologist. The patient’s history of Eisenmengers, as well as cardiac function is expected to worsen with time and we are predicting the same for the hoarseness. Current imaging shows a large partially calcified PDA with finding of PAH with enlarged central pulmonary arteries, corresponding to our aforementioned beliefs on worsening respiratory and cardiac symptoms. This is an expected outcome for our patient with PDA. In addition to Nakahira and colleagues’ [7] case study, we think our case study supports that long-standing PDA causes enlarged central pulmonary arteries which result in compression on the left recurrent laryngeal nerve causing hoarseness for our patient. It can, therefore, be assumed that the underlying Eisenmenger’s syndrome is the driving factor to cause PDA, and the subsequent enlargement of pulmonary arteries produced a mass effect on recurrent laryngeal nerves, resulting in voice hoarseness.

Eisenmenger syndrome is the constellation of symptoms that arise from a congenital heart defect with a systemic-to-pulmonary shunt which generally becomes symptomatic in children before puberty but may develop in adolescence or early adulthood [14]. The pathophysiological changes are arising due to pulmonary arterial hypertension resulting in complex, unrepaired congenital heart disease as early as the first decade of life. The usual life expectancy is between 20 and 30 years [15], and with current medical advances and early diagnosis, some patients survive into the sixth decade of life [16]. Mortality associated with Eisenmenger syndrome occurring early on in life occurs due to thromboembolic events and in relation to both cardiac and non-cardiac causes. However, chronic cardiac conditions increased with age [17]. In adults, the chronic slow progressive hypoxemia with central cyanosis is thought to be the cause of the complex and multisystemic disorder, including coagulation disorders (bleeding complications and paradoxical embolisms), renal dysfunction, hypertrophic osteoarthropathy, heart failure, reduced quality of life and premature death [18]. The age of the patient in our case study is 77 years compared to the mean range of 20–30 years and has shown many symptoms associated with Eisenmenger’s syndrome including PDA which resulted in a mass effect causing voice hoarseness. As an increasing number of patients with Eisenmenger syndrome survive [19] due to advances in cardiac care, an appreciation of the pathophysiology and sequelae of symptoms including hoarseness of voice is imperative in the field of otolaryngology.

## Conclusion

In keeping with our case study, we believe that slow worsening of cardiac function subsequently led to the dilatation of the pulmonary arteries in a 77-year-old woman with a history of long-standing PDA resulting in a mass effect on the left recurrent laryngeal nerve, causing hoarseness of voice. Patients with Eisenmenger’s Syndrome need to be followed for potential recurrent laryngeal nerve paralysis.

## Data Availability

Data sharing is not applicable to this article as no datasets were generated or analysed during the current study.

## Abbreviations

COPD: Chronic obstructive pulmonary disease
CT: Computed tomography
PAH: Pulmonary arterial hypertension
PDA: Patent ductus arteriosus
